# Supplementary material for: Design rules for catalysis in single-particle plasmonic nanogap reactors with precisely aligned molecular monolayers
Source: Nat Commun. 2024 Oct 25;15:9220. doi: 10.1038/s41467-024-53544-3 (PMC11511967; doi:10.1038/s41467-024-53544-3)
Supplement: Supplementary file 1 — Supplementary Information [file 41467_2024_53544_MOESM1_ESM.pdf]

Supplementary Information

Design Rules for Catalysis in Single-particle Plasmonics Nanogap Reactors with Precisely Aligned Molecular Monolayers

Gyeongwon Kang,[1,2] Shu Hu,[1] Chenyang Guo,[1] Rakesh Arul,[1] Sarah M. Sibug-Torres,[1] Jeremy J. Baumberg*,[1]

[1]Nanophotonics Centre, Department of Physics, Cavendish Laboratory, University of Cambridge, Cambridge, CB3 0HE, UK

[2]Department of Chemistry, Kangwon National University, Chuncheon, 24341, South Korea

*e-mail: jjb12@cam.ac.uk

Supplementary Figures

Suppl. Fig. 1 | TEM images of 80 nm Au@Pd NPs 3

Suppl. Fig. 2 | Cyclic voltammograms of Au (0 ML Pd) and Au@Pd NPs with different coverages of Pd 4

Suppl. Fig. 3 | Dark-field images of NRs 5

Suppl. Fig. 4 | SEM images of NRs 5

Suppl. Fig. 5 | FDTD simulations results for NRs 6

Suppl. Fig. 6 | Time-series SERS spectra of 4-BPT from individual NRs in ambient conditions 7

Suppl. Fig. 7 | Time-series SERS spectra of NC-BTP from individual NRs in ambient conditions 7

Suppl. Fig. 8 | Laser-power dependent SERS spectra 8

Suppl. Fig. 9 | DFT-calculated vibrational modes 9

Suppl. Fig. 10 | SERS spectra from each NR type in solution of phenylboranic acid (PBA) and K_2CO_3 9

Suppl. Fig. 11 | Lorentzian peak fitting results for NRs 10

Suppl. Fig. 12 | Progress of 1550 cm^{-1} mode intensity during the reaction 11

Suppl. Fig. 13 | SEM images MLaggs 11

Suppl. Fig. 14 | Averaged SERS of MLaggs before and after reaction 12

Suppl. Fig. 15 | Time-series SERS spectra colormaps from MLaggs during reaction 12

Suppl. Fig. 16 | Lorentzian peak fitting results for MLaggs 13

Suppl. Fig. 17 | SERS spectra with 785 nm laser excitation 13

Suppl. Fig. 18 | Peak analysis results of Au and Pd MLaggs at different laser powers 14

Suppl. Fig. 19 | Schematic top views of coverages of 4-BTP and NC-BPT 15

Suppl. Fig. 20 | DFT-calculated projected density of states (PDOS) of surface atoms 16

Suppl. Fig. 21 | Electrochemical characterization of Au@Pd films 16

Supplementary Tables

Suppl. Table 1 | SERS intensity ratio of 1550 cm^{-1} mode of NC-BPT to 1532 cm^{-1} mode of 4-BTP. (I_{1550}/I_{1532}) .. 17

Suppl. Table 2 | SERS intensity ratio of 1572 cm^{-1} mode of NC-BPT to 1532 cm^{-1} mode of 4-BTP. (I_{1572}/I_{1532}) .. 17

Suppl. Table 3 | SERS intensity ratio of 2255 cm^{-1} mode of NC-BPT to 1532 cm^{-1} mode of 4-BTP. (I_{2255}/I_{1532}) . 18

Suppl. Table 4 | Estimated number of adsorbed 4-BPT and NC-BPT molecules within each nanogap............ 19

Supplementary Notes

Suppl. Note 1 | SERS intensity ratio of product modes to 1532 cm^{-1} mode of 4-BTP 17

Suppl. Note 2 | Information on MLaggs 18

Suppl. Note 3 | Fitting dynamics of SERS peak intensity ratio (R_{21} and R_{31}) in Figure 4a-d.............................. 18

Suppl. Note 4 | Estimation of number of reactant and product molecules in the gap 19

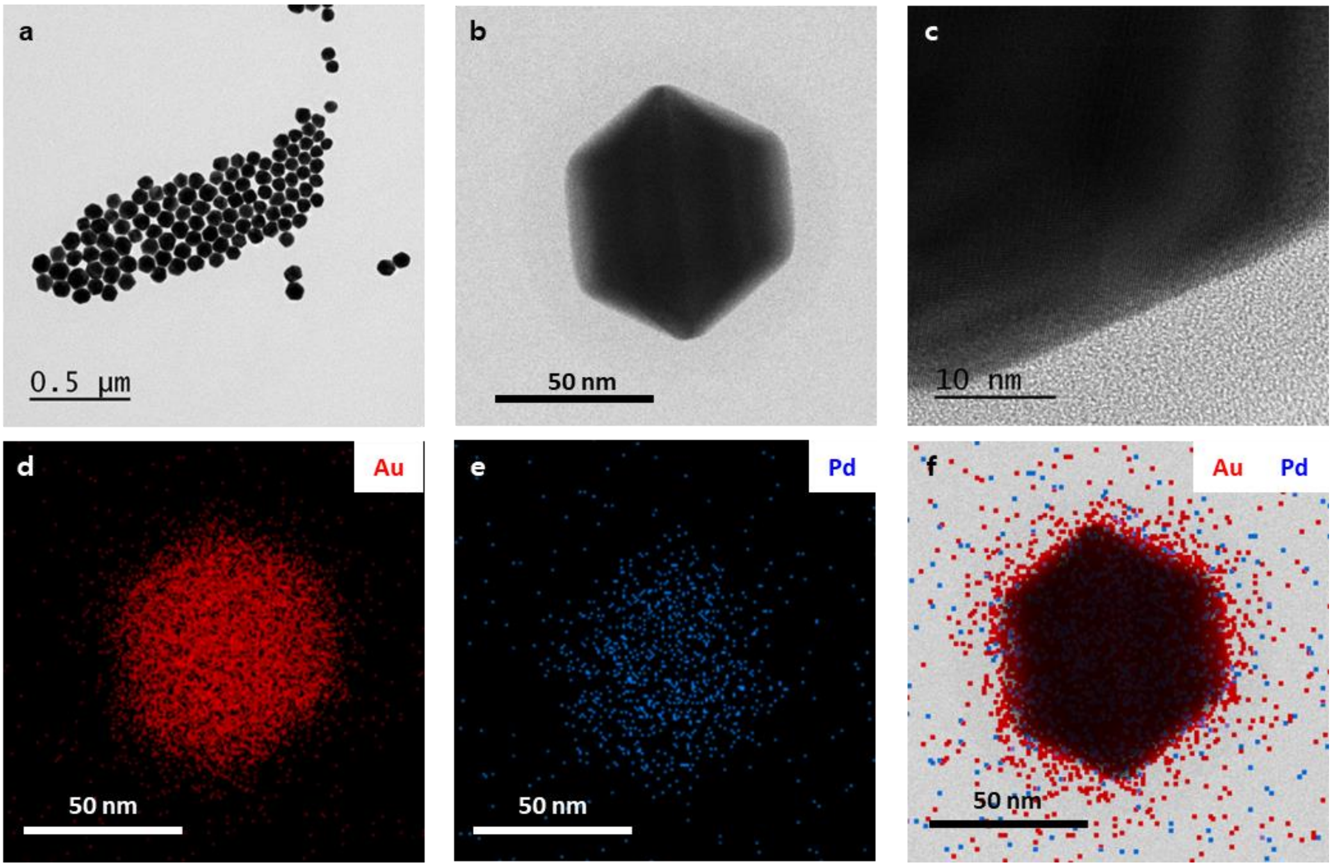

Suppl. Fig. 1 | TEM images of 80 nm Au@Pd NPs. (a) A cluster of Au@Pd NPs with narrow size distribution. **(b)** Low and **(c)** high resolution TEM images of single Au@Pd NP. EDS maps of **(d)** Au and **(e)** Pd and **(f)** an overlaid image of (b), (d), and (e).

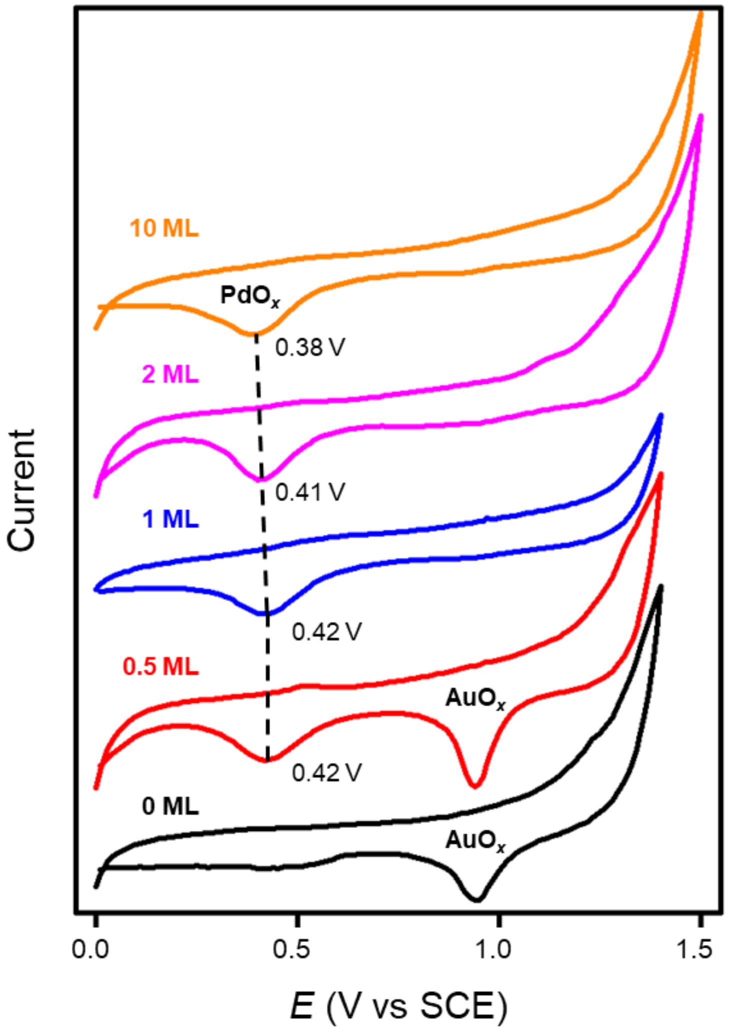

Suppl. Fig. 2 | Cyclic voltammograms of Au (0 ML Pd) and Au@Pd NPs with different coverages of Pd. Coverages of Pd on Au NP are 0.5, 1, 2 and 10 ML. (bottom to top) (Scan rate = 10 mV s^{-1}, 0.1 M H_2SO_4) NPs are centrifuged and redispersed in water twice to remove extra capping ligands. Precipicated NPs are dropcasted onto a glass carbon electrode (working electrode) and then dried for electrochemical measurement. A Pt wire and Ag/AgCl are used for counter and refernece electrodes, respectively. Potential E is plotted with respect to saturated calomel electrode (SCE) potential.

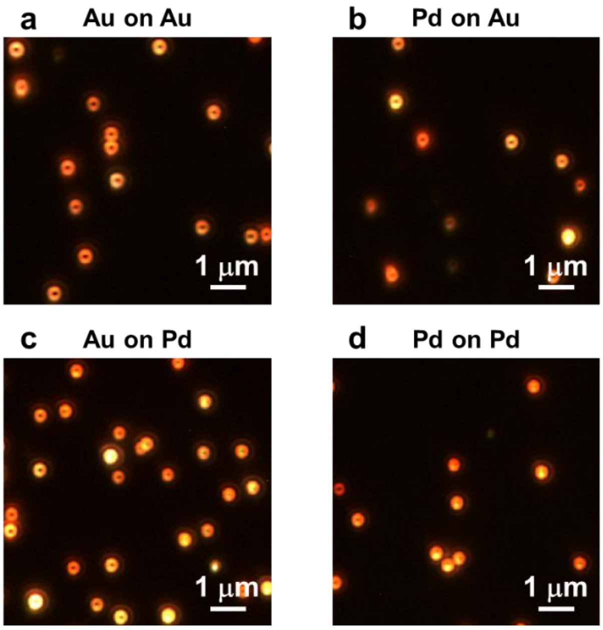

Suppl. Fig. 3 | Dark-field images of NRs. (a) Au-on-Au, **(b)** Pd-on-Au, **(c)** Au-on-Pd, and **(d)** Pd-on-Pd.

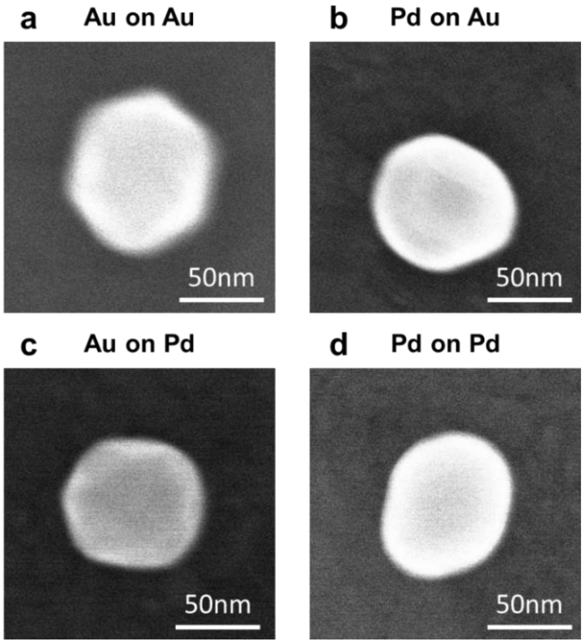

Suppl. Fig. 4 | SEM images of NRs. (a) Au-on-Au, **(b)** Pd-on-Au, **(c)** Au-on-Pd, and **(d)** Pd-on-Pd.

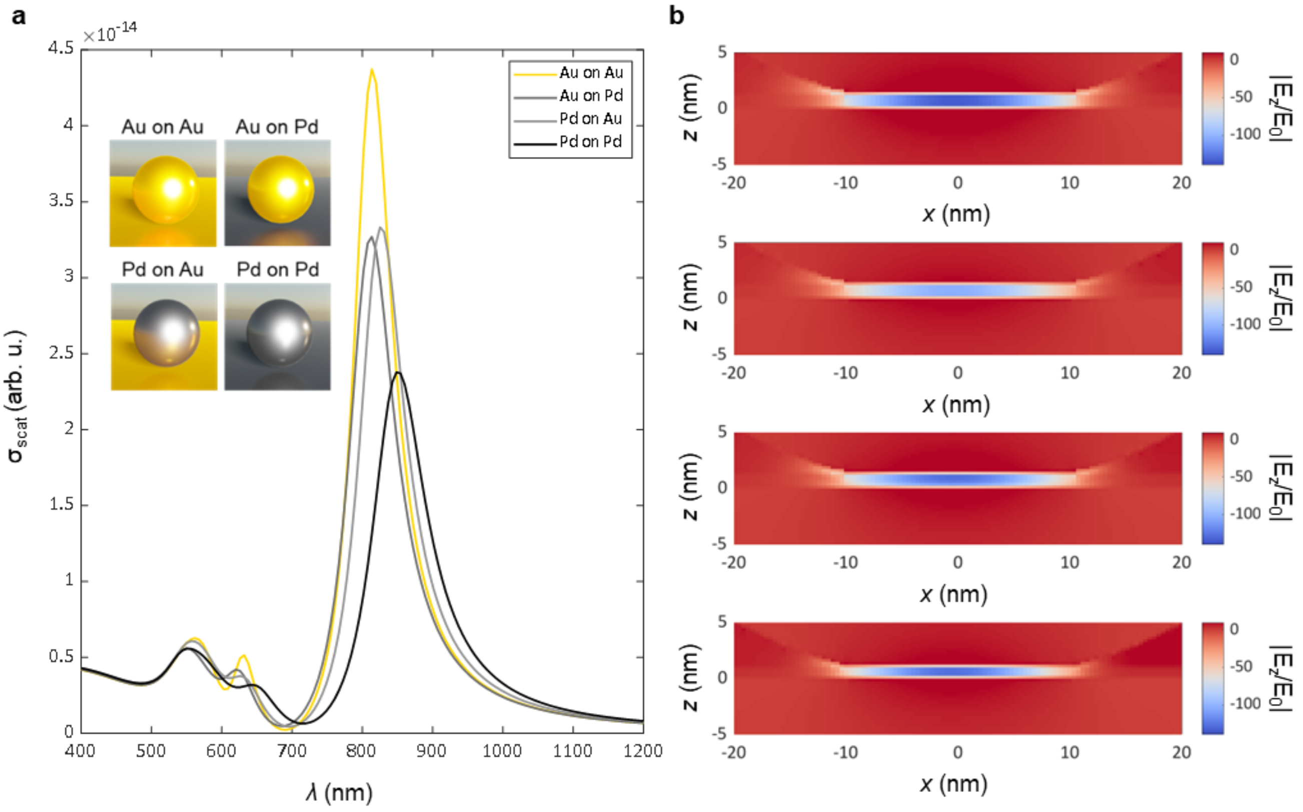

Suppl. Fig. 5 | FDTD simulations results for NRs. (a) Optical scattering cross section with a dielectric gap (*n* = 1.5 and *d* = 1.2 nm), and a circular facet diameter of 20 nm. 0.33-nm-thick Pd layer is used to model Pd ML. **(b)** Near-field distribution of $|E_z/E_0|$ of nanogap within each NR at its dominant plasmonic resonance.

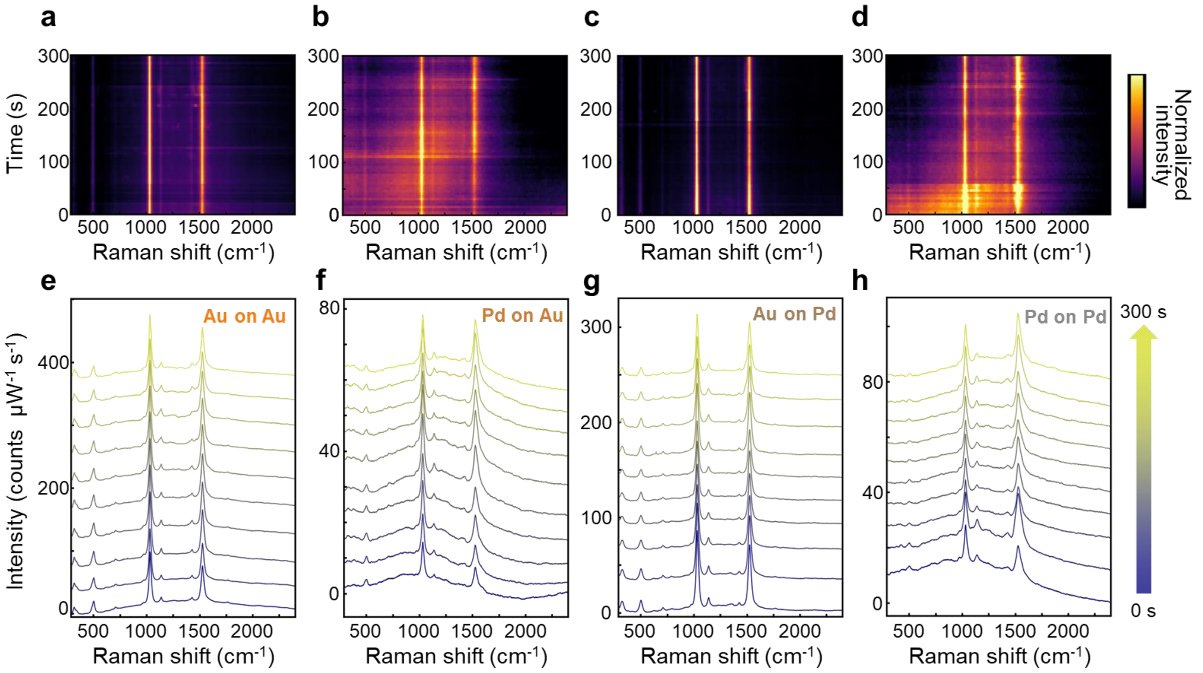

Suppl. Fig. 6 | Time-series SERS spectra of 4-BPT from individual NRs in ambient conditions. Representative SERS spectra colormaps of **(a)** Au-on-Au, **(b)** Pd-on-Au, **(c)** Au-on-Pd, and **(d)** Pd-on-Pd NR, respectively. Shown in each map are 300 spectra of 1 s integration time. **(e-h)** Stacked SERS spectra acquired every 30 s from colormaps above. Each SERS spectrum is measured with acquisition time of 1 s and 0.1 mW of 633 nm laser power.

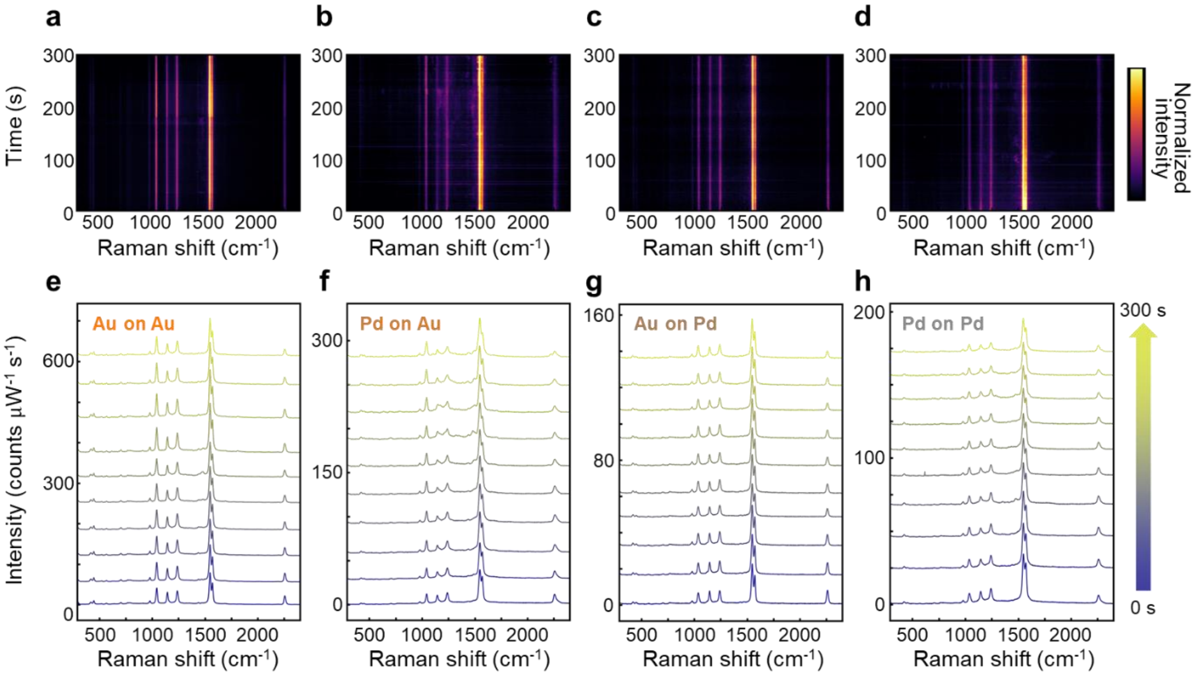

Suppl. Fig. 7 | Time-series SERS spectra of NC-BTP from individual NRs in ambient conditions. Representative SERS spectra colormaps of **(a)** Au-on-Au, **(b)** Pd-on-Au, **(c)** Au-on-Pd, and **(d)** Pd-on-Pd NR, respectively. Shown in each map are 300 spectra of 1 s integration time. **(e-h)** Stacked SERS spectra acquired every 30 s from colormaps above. Each SERS spectrum is measured with acquisition time of 1 s and 0.05 mW of 633 nm laser power.

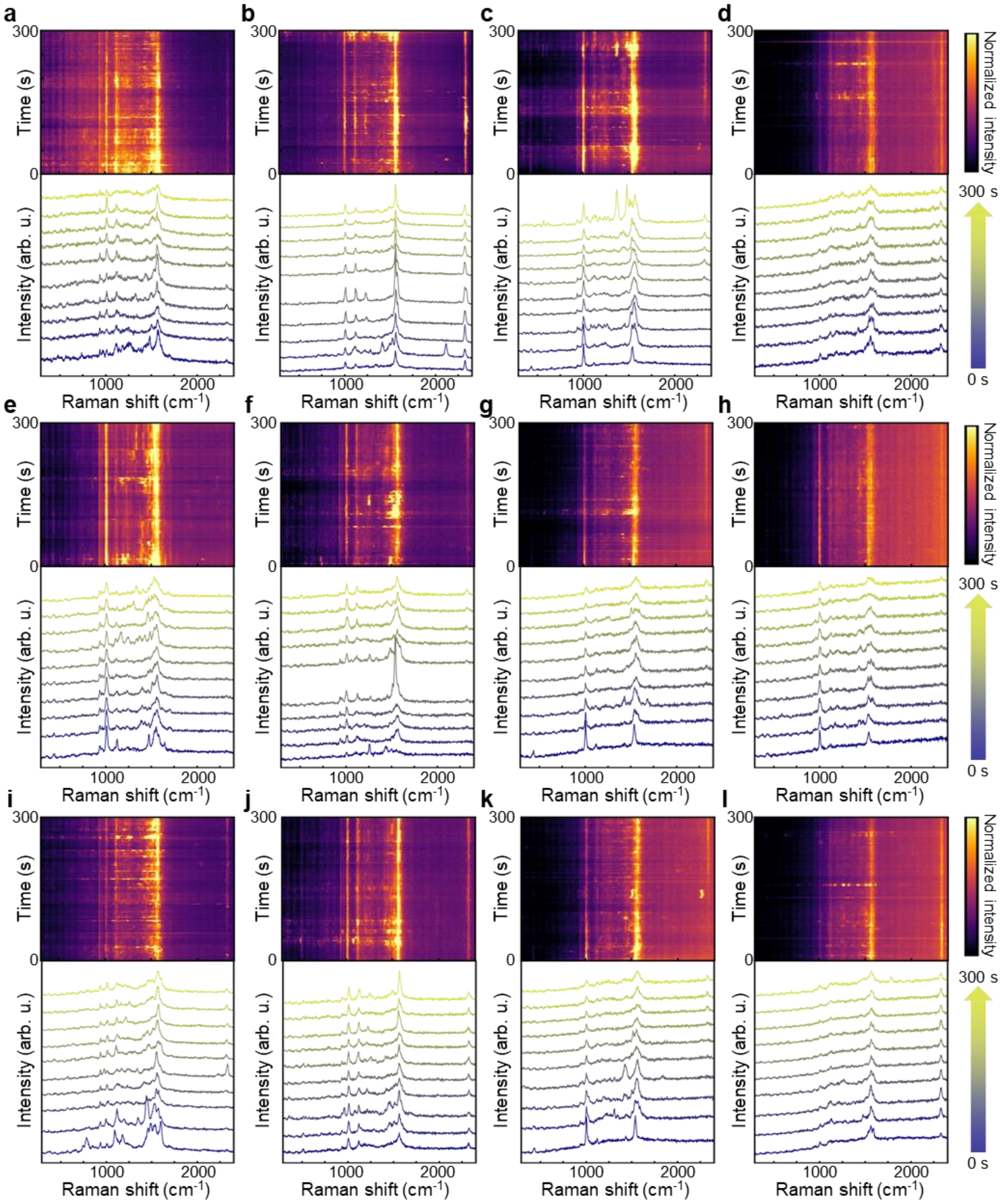

Suppl. Fig. 8 | Laser-power dependent SERS spectra. Colourmaps of SERS spectra (300 successive spectra @ 1 s integration times) using **(a,e,i)** Au-on-Au, **(b,f,j)** Pd-on-Au, **(c,g,k)** Au-on-Pd, and **(d,h,l)** Pd-on-Pd NRs. (Laser powers: a,b,c,d - 0.2 mW, e,f,g,h - 0.3 mW, I,j,k,l - 0.4 mW) iIndividual 1 s spectra every 30 s are shown as stacked plot below each colourmap.

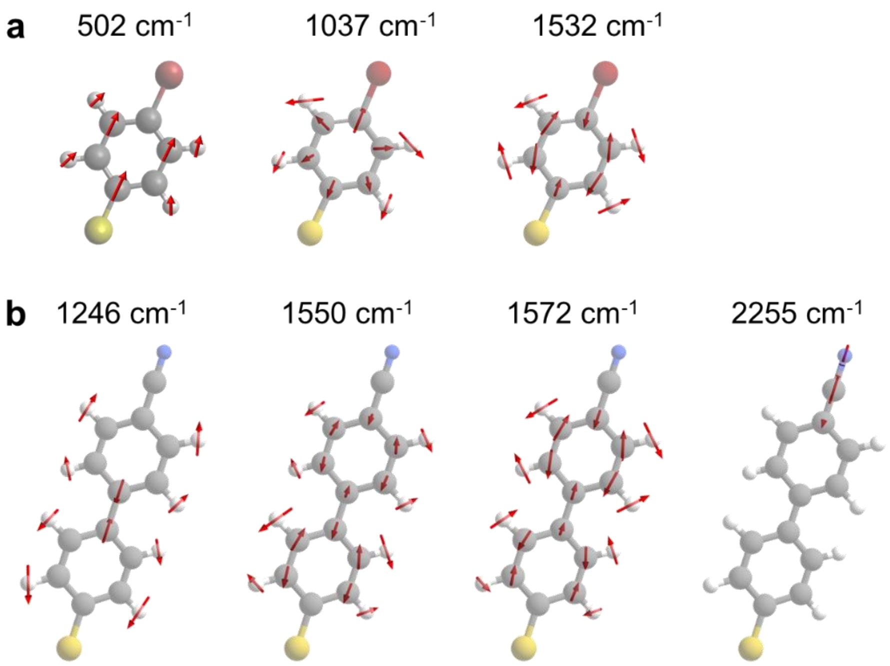

Suppl. Fig. 9 | DFT-calculated vibrational modes. Dominant modes in the SERS are selected for visualization for **(a)** 4-BTP and **(b)** NC-BPT.

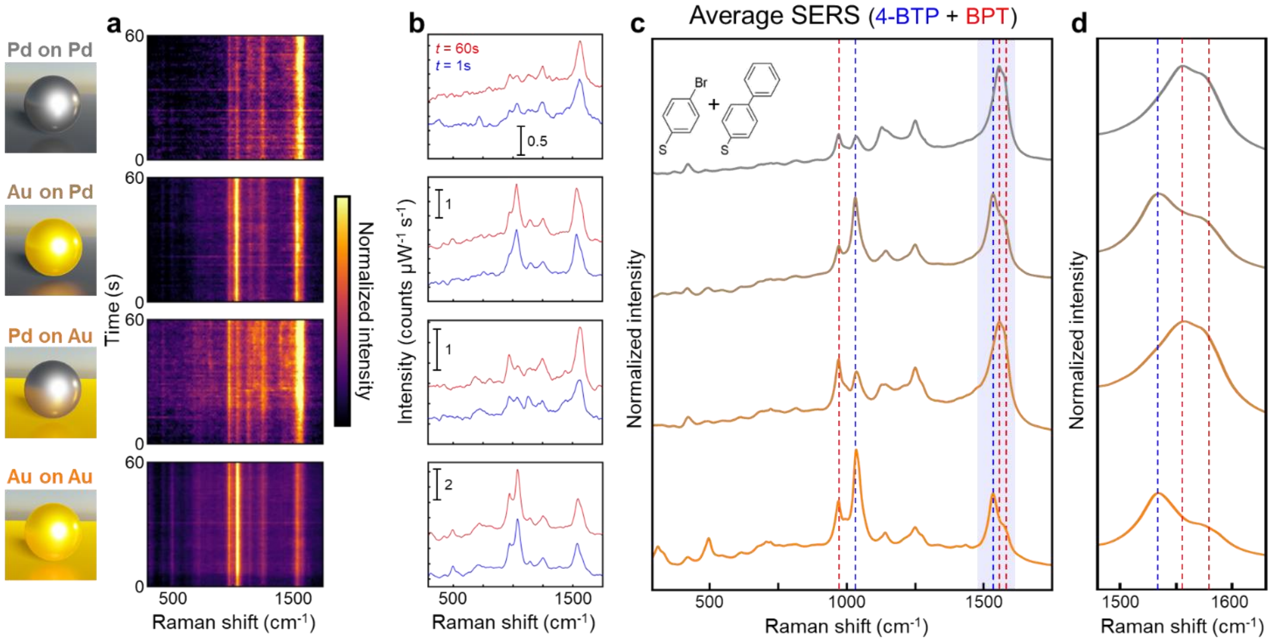

Suppl. Fig. 10 | SERS spectra from each NR type in solution of phenylboranic acid (PBA) and K2CO3. **(a)** Colourmaps of repeated SERS spectra (60 frames @ 1 s integration times). **(b)** Initial (*t* = 1 s, blue) and final (*t* = 60 s, red) SERS spectra from colourmaps in (a). **(c)** SERS spectra normalized by maximum intensity in solution during 60 s for each NR type. **(d)** SERS spectra of blue-shaded region in (c). From bottom to top: Au-on-Au, Pd-on-Au, Au-on-Pd, and Pd-on-Pd NRs. Note that the concentration of PBA in the solution is 67 mM (10 times higher than cPBA solution) to observe saturation of the reaction within a reasonable amount of time.

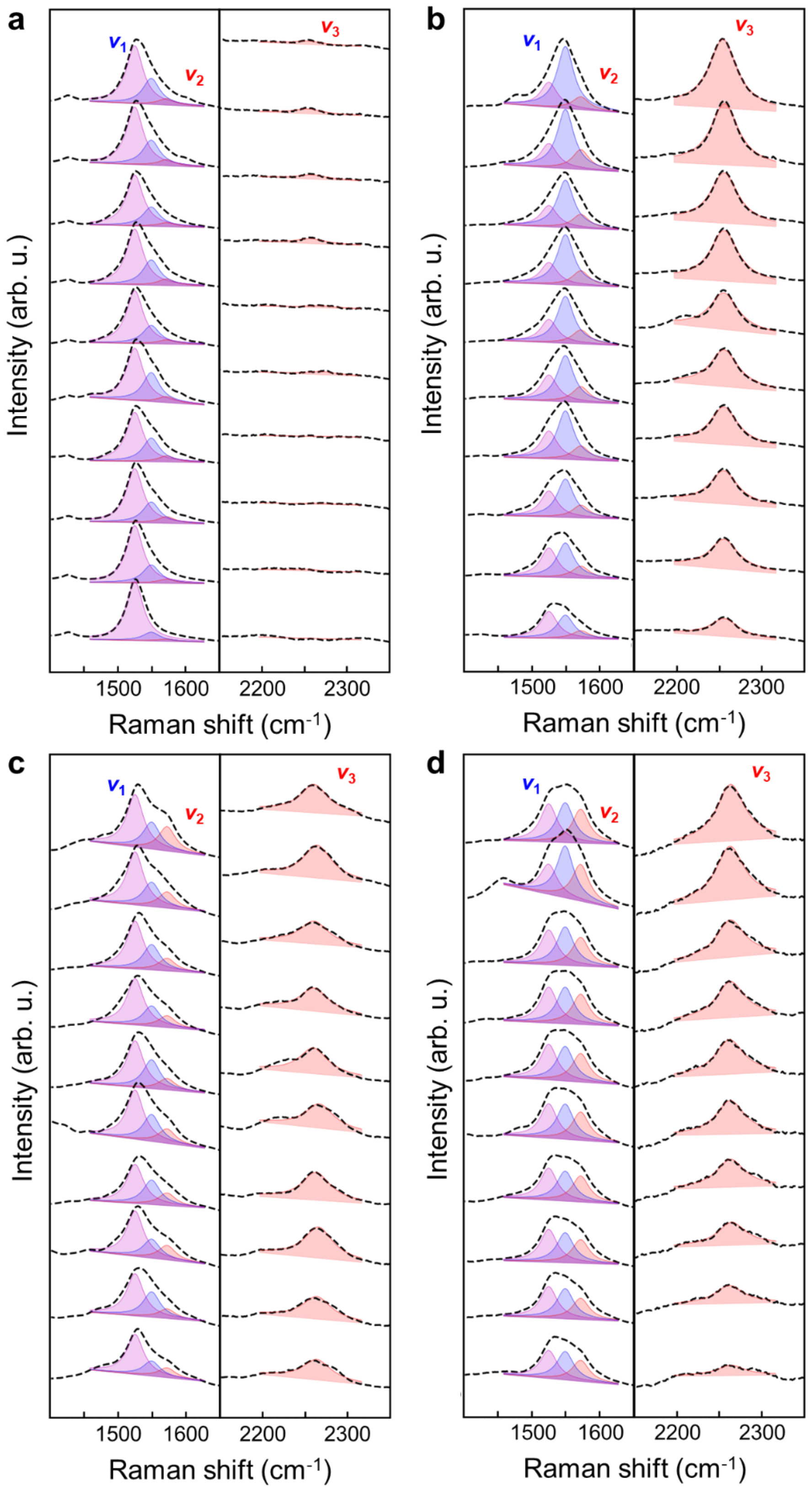

Suppl. Fig. 11 | Lorentzian peak fitting results for NRs. Fitting results for **(a)** Au-on-Au, **(b)** Pd-on-Au, **(c)** Au-on-Pd, and **(d)** Pd-on-Pd NRs.

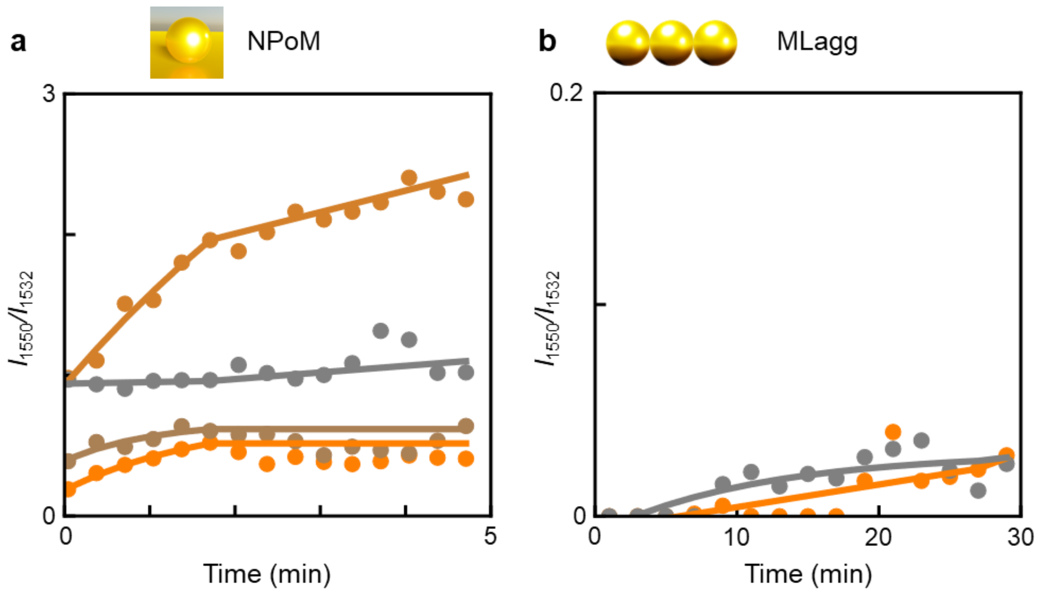

Suppl. Fig. 12 | Progress of 1550 cm^{-1} mode intensity during the reaction. Peak intensity ratio of 1550 cm^{-1} NC-BPT mode to 1532 cm^{-1} mode of 4-BTP for **(a)** NRs (Au-on-Au: orange, Pd-on-Au: darker orange, Au-on-Pd: brown, and Pd-on-Pd: grey) and **(b)** MLaggs (Au: orange, and Pd: grey).

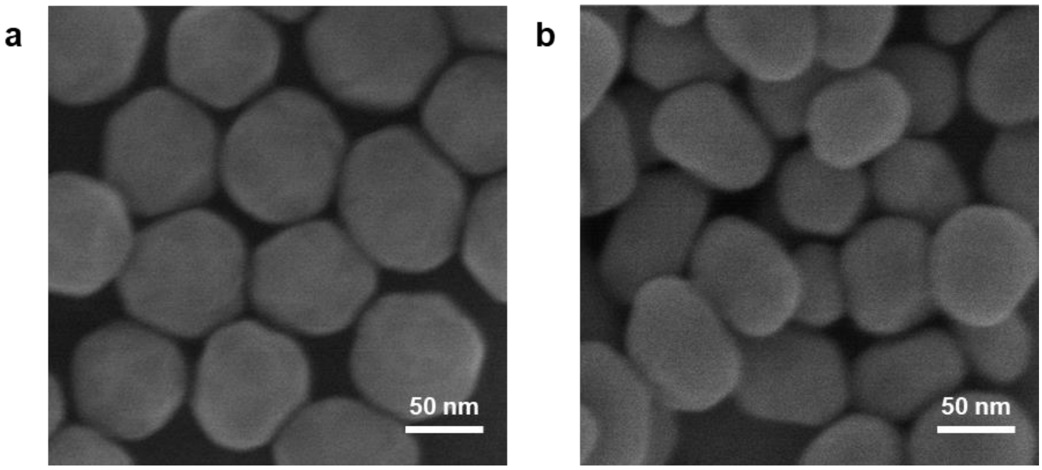

Suppl. Fig. 13 | SEM images MLaggs. (a) Au and **(b)** Pd MLaggs.

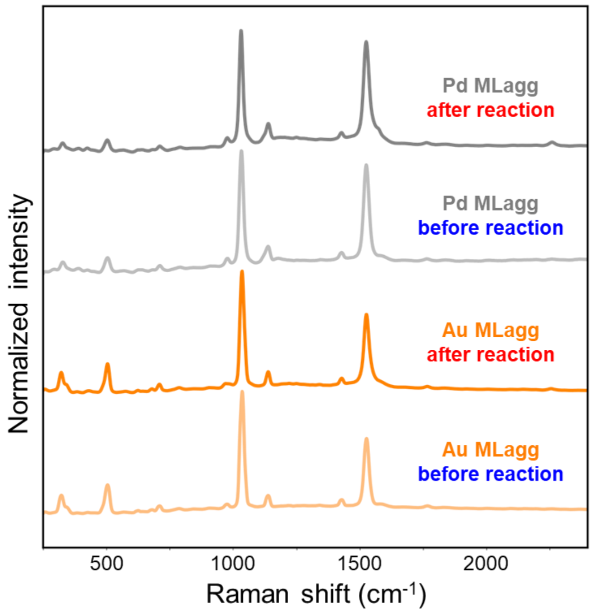

Suppl. Fig. 14 | Averaged SERS of MLaggs before and after reaction. Au (orange) and Pd (grey) MLaggs before (brighter curves) and after (darker curves) reaction, under laser illumination with 0.4 mW of 633 nm laser power.

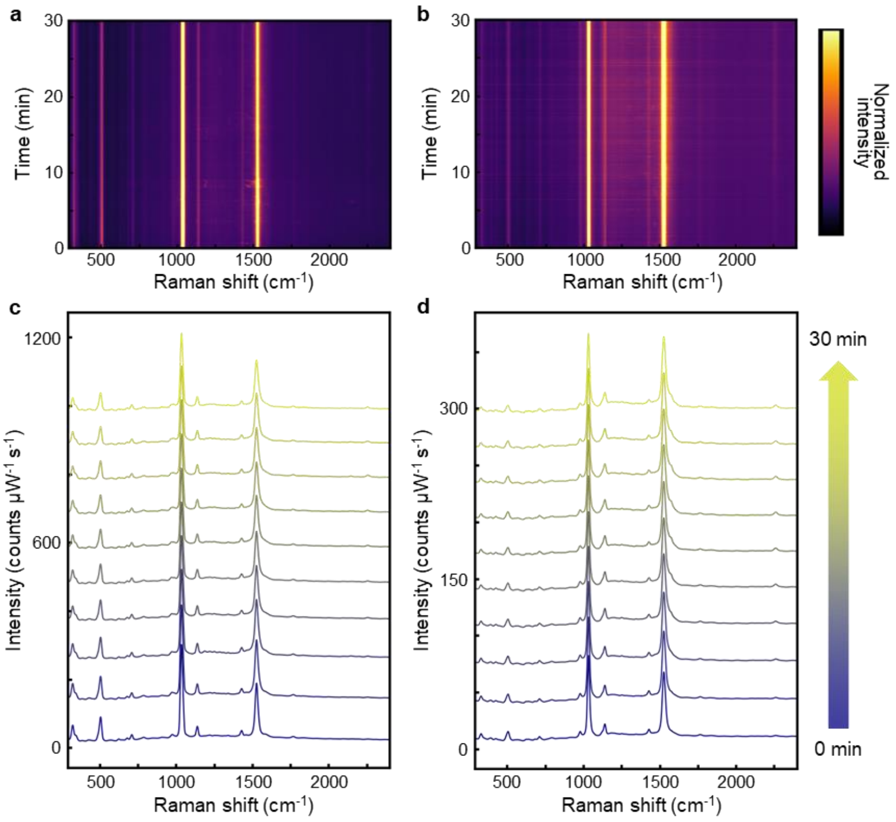

Suppl. Fig. 15 | Time-series SERS spectra colormaps from MLaggs during reaction. SERS spectra colormaps of **(a)** Au and **(b)** Pd MLaggs in aqueous solution of CPBA and K_2CO_3, respectively. (300 spectra with 1 s integration time) **(c,d)** Stacked SERS spectra taken every 3 min, from colormaps above. Each SERS spectrum is measured with acquisition time of 1 s and 0.4 mW of 633 nm laser power.

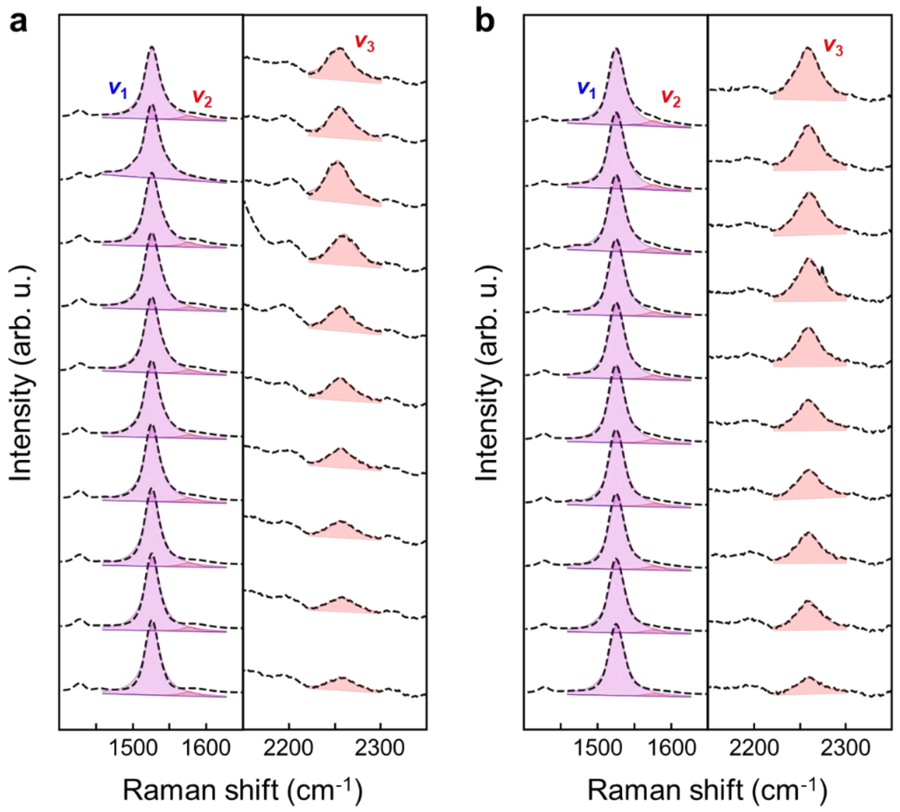

Suppl. Fig. 16 | Lorentzian peak fitting results for MLaggs. Fitting results for **(a)** Au and **(b)** Pd MLaggs.

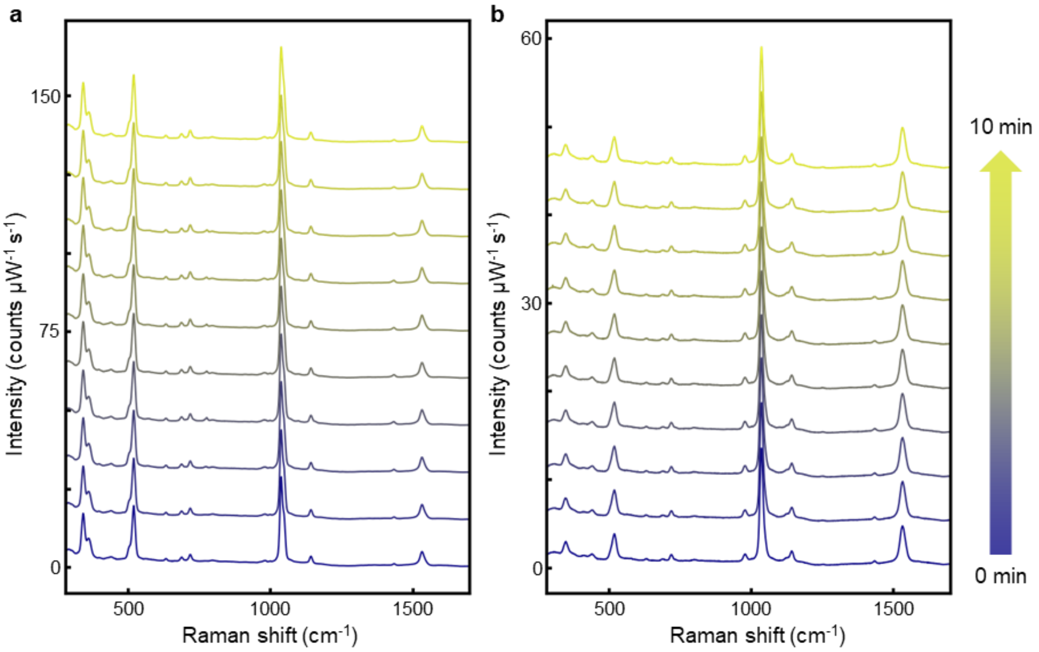

Suppl. Fig. 17 | SERS spectra with 785 nm laser excitation. Stacked SERS spectra of **(a)** Au and **(b)** Pd MLaggs acquired every minute. Each SERS spectrum is measured with acquisition time of 1 s and 0.2 mW of laser power.

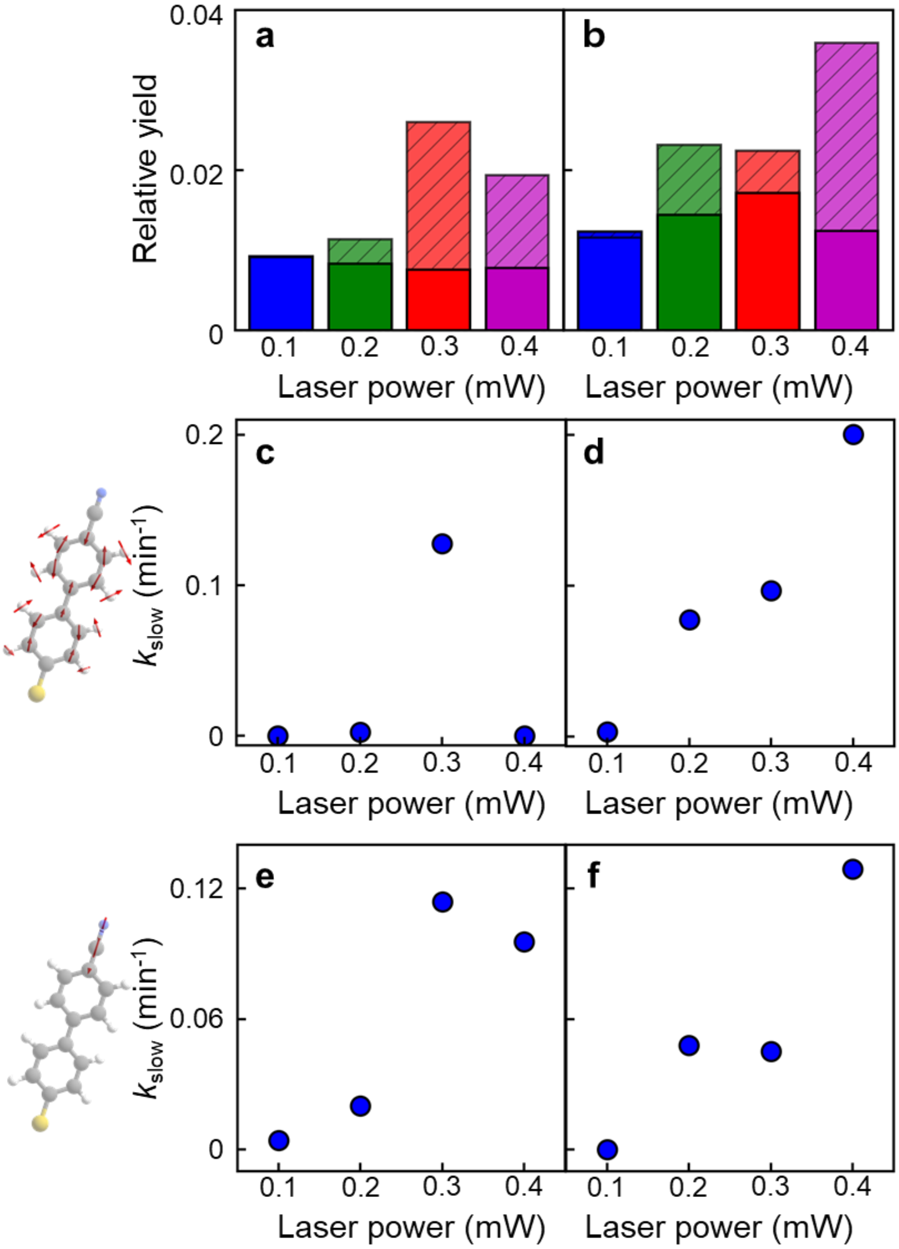

Suppl. Fig. 18 | Peak analysis results of Au and Pd MLaggs at different laser powers. (a, b) Catalytic conversion yields for laser illumination after t = 0 (darker bars) and t = 10 min (lighter bars with hatched lines). Slow rate constants (k_{slow}) obtained from the peak intensity ratio of **(c, d)** 1572 cm^{-1} (v_2) and **(e, f)** 2255 cm^{-1} (v_3) NC-BPT modes to the 1532 cm^{-1} mode of 4-BTP (v_1).

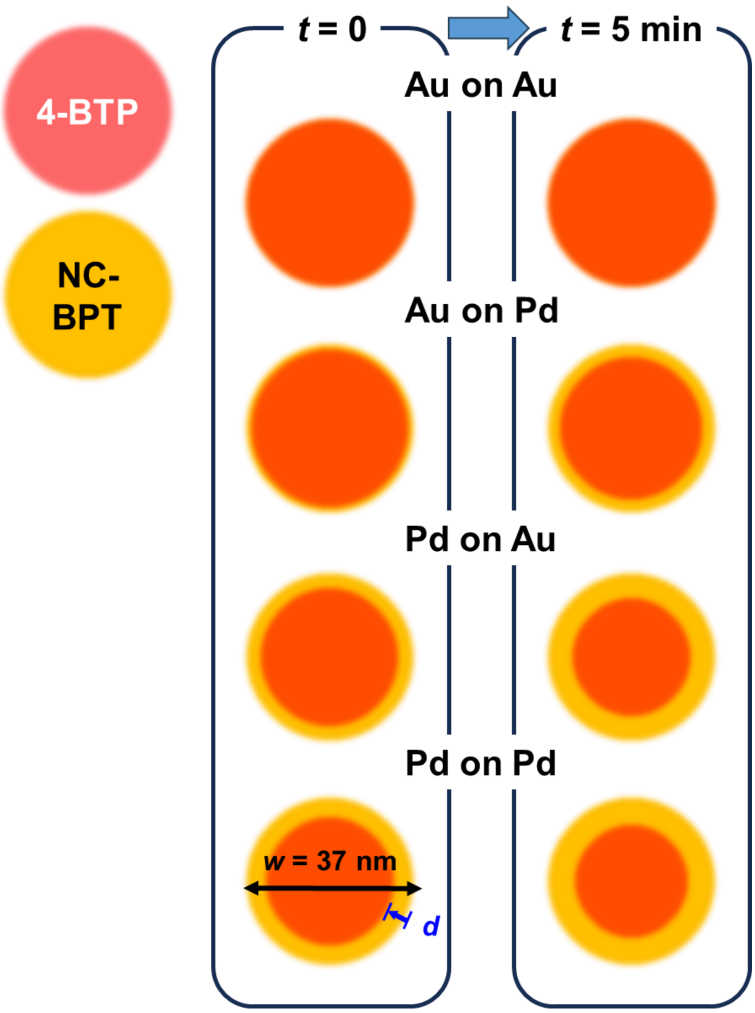

Suppl. Fig. 19 | Schematic top views of coverages of 4-BTP and NC-BPT. Each NR gap beneath a NP with diameter of 80 nm and facet size of 37 nm is visualized. Red and yellow areas indicate the estimated adsorption surface area covered by 4-BTP and NC-BPT, respectively. See Suppl. Note 4 for details of the calculation of number of adsorbed molecules, adsorption area, and radial diffusion length *d* to reach unreacted molecules.

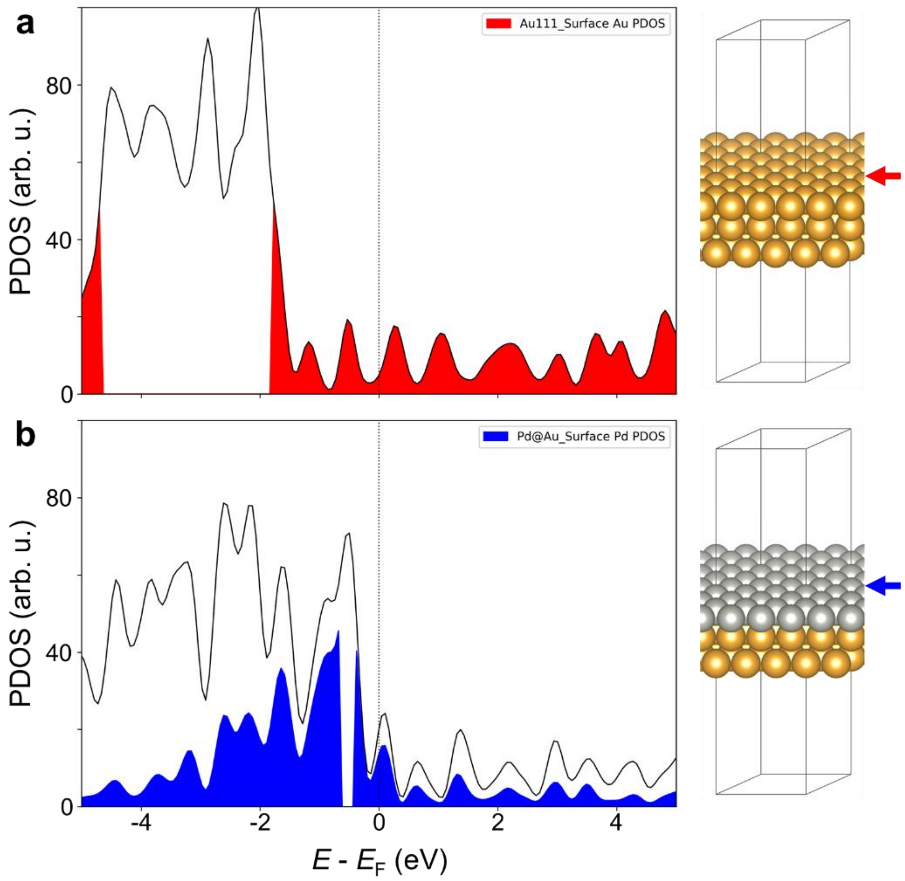

Suppl. Fig. 20 | DFT-calculated projected density of states (PDOS) of surface atoms. (a) PDOS of Au(111) slab with PDOS of Au top layer atoms highlighted in red. **(b)** PDOS of Pd ML on Au(111) slab with PDOS of Pd top layer atoms highlighted in blue. Optimized DFT geometries used to calculated PDOS are shown to right.

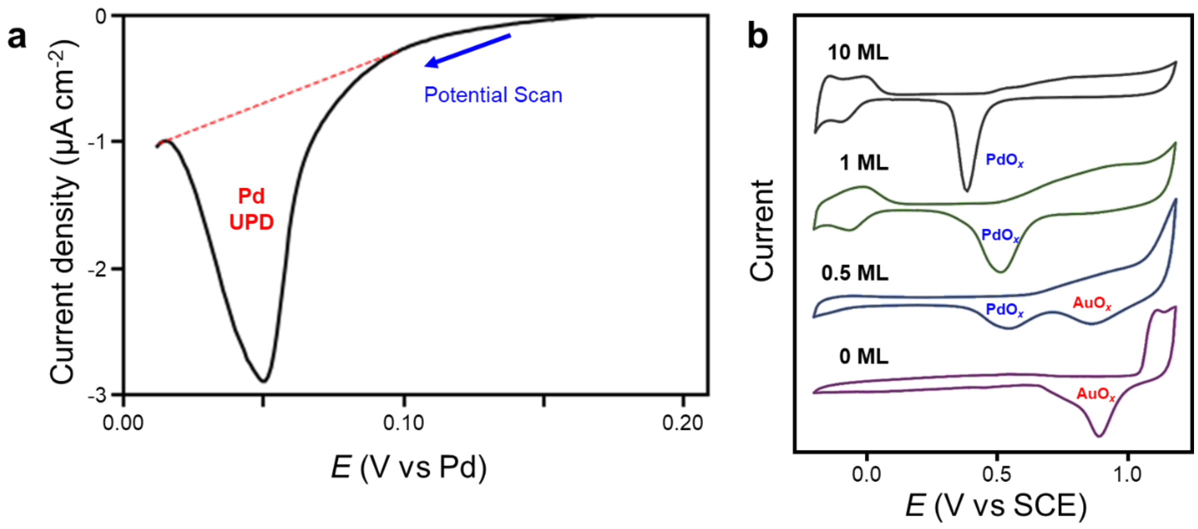

Suppl. Fig. 21 | Electrochemical characterization of Au@Pd films. (a) Linear potential sweep voltammetry during the underpotential deposition of Pd on a template-stripped Au film. **(b)** Cyclic voltammograms of Au (0 ML Pd) and Au@Pd films with different coverages of Pd of 0.5, 1, and 10 ML. (bottom to top)

Suppl. Note 1 | SERS intensity ratio of product modes to 1532 cm-1 mode of 4-BTP

Time (s)	Au on Au	Au on Pd	Pd on Au	Pd on Pd	Time (min)	Au MLagg	Pd MLagg
20	0.26113	0.386701	1.084555	0.898983	2	0	0
40	0.288821	0.495471	1.181694	0.910547	4	0	0
60	0.380646	0.539038	1.761205	0.923559	6	0	0
80	0.432901	0.652976	1.629218	0.952425	8	0.001194	0
100	0.515225	0.599373	1.827035	0.951686	10	0.004969	0.015167
120	0.489598	0.618609	1.950762	0.967639	12	0	0.020914
140	0.374129	0.641461	1.887555	1.107547	14	0	0.014138
160	0.403345	0.610402	2.045449	1.013488	16	0	0.019995
180	0.366371	0.524386	2.3868	0.991351	18	0	0.017883
200	0.309289	0.408142	1.941263	0.992575	20	0.016741	0.027917
220	0.381961	0.439816	2.139522	1.251857	22	0.03979	0.031778
240	0.377866	0.493668	2.313413	1.437537	24	0.016722	0.03578
260	0.422491	0.3293	2.469201	1.070471	26	0.018628	0.021531
280	0.399386	0.587898	2.23587	0.974127	28	0.022118	0.012127
300	0.43318	0.675335	2.675726	1.013364	30	0.028758	0.024735

Suppl. Table 1 | SERS intensity ratio of 1550 cm^{-1} mode of NC-BPT to 1532 cm^{-1} mode of 4-BTP. (I_{1550}/I_{1532})

Time (s)	Au on Au	Au on Pd	Pd on Au	Pd on Pd	Time (min)	Au MLagg	Pd MLagg
20	0.068664	0.126553	0.40356	0.657528	2	0.060362	0.045237
40	0.06352	0.26019	0.443556	0.698459	4	0.059094	0.051451
60	0.092291	0.220865	0.553991	0.702741	6	0.053066	0.057839
80	0.121574	0.277845	0.520676	0.765237	8	0.05782	0.062334
100	0.113431	0.369584	0.540099	0.7504	10	0.039383	0.072475
120	0.105753	0.223209	0.612962	0.778925	12	0.057413	0.08679
140	0.073591	0.241769	0.695973	0.823359	14	0.061794	0.101733
160	0.165134	0.257195	0.524494	0.860374	16	0.055755	0.101365
180	0.113631	0.266213	0.597636	0.796682	18	0.056645	0.109455
200	0	0.23686	0.652361	0.817445	20	0.060673	0.112514
220	0.06166	0.264662	0.582032	0.752829	22	0.056673	0.108705
240	0.110032	0.314811	0.809172	1.034952	24	0.056867	0.112462
260	0.078957	0.261636	0.711084	0.858459	26	0.056838	0.105871
280	0.072411	0.449428	0.675534	0.807838	28	0.053313	0.095951
300	0.087819	0.370822	0.83354	0.944194	30	0.059167	0.10522

Suppl. Table 2 | SERS intensity ratio of 1572 cm^{-1} mode of NC-BPT to 1532 cm^{-1} mode of 4-BTP. (I_{1572}/I_{1532})

Time (s)	Au on Au	Au on Pd	Pd on Au	Pd on Pd	Time (min)	Au MLagg	Pd MLagg
20	0.004027	0.071122	0.245289	0.113669	2	0.008098	0.016731
40	0.009036	0.101684	0.299669	0.142407	4	0.010247	0.02083
60	0.012485	0.10431	0.424864	0.168004	6	0.013115	0.023968
80	0.002909	0.098901	0.389789	0.230993	8	0.019746	0.029426
100	0.015286	0.107397	0.432609	0.193572	10	0.018621	0.033363
120	0.012218	0.093134	0.418386	0.229713	12	0.027288	0.040367
140	0.009985	0.091416	0.502599	0.276541	14	0.02709	0.05114
160	0.016366	0.141604	0.445411	0.262617	16	0.023554	0.054859
180	0.019177	0.093861	0.621348	0.291334	18	0.028628	0.057575
200	0	0.084428	0.595984	0.25809	20	0.033696	0.059821
220	0.011791	0.086692	0.680947	0.262157	22	0.053844	0.058829
240	0.013683	0.080339	0.695447	0.359251	24	0.04533	0.056791
260	0.018741	0.10406	0.733574	0.360073	26	0.039697	0.054029
280	0.018758	0.096265	0.73829	0.352367	28	0.035143	0.053028
300	0.029081	0.156132	0.989262	0.340377	30	0.035823	0.05473

Suppl. Table 3 | SERS intensity ratio of 2255 cm^{-1} mode of NC-BPT to 1532 cm^{-1} mode of 4-BTP. (I_{2255}/I_{1532})

Suppl. Note 2 | Information on MLaggs

The detailed characterization of MLaggs have been reported from our group previously.[1,2] In the SI of ref. [1], experimental and theoretical (FDTD) data on different layers of MLagg show that our 633 nm laser directly couples into the nanogaps, producing SERS from only molecules within the gaps. Further imaging (SEM) and optical characterizations (dark-field spectra) presented in the SI of ref. [2] show MLaggs preserve gap sizes of <1 nm while forming close-packed nanoparticles arrays with dense nanogaps. While the dominant plasmon resonances of the MLaggs is tuned beyond λ > 1 µm, higher order resonances are observed around 633 nm.

Suppl. Note 3 | Fitting dynamics of SERS peak intensity ratio (R_{21} and R_{31}) in Figure 4a-d

In the first stage of the catalysis, rapid reaction occurs upon hot-carrier generation if there is excess CPBA in the gap. The reaction yield thus follows a 1st order kinetics,

$$R = R_0\left(1 - \mathrm{e}^{-k_{\mathrm{fast}}t}\right) + C \qquad (1)$$

where C is the initial yield from reactions occurring faster than the measurement time scale (1 s).

After consumption of CPBA in the gap during this rapid 1st order reaction, diffusion-limited reaction proceeds which is much slower due to the limited catalytic sites in the gap. This gives 0th order kinetics as the reaction is not dependent on the reactant population any more, and the reaction yield is

$$R = k_{\mathrm{slow}}t + D \qquad (2)$$

Here the constant D is the yield from initial 1st order reaction.

Suppl. Note 4 | Estimation of number of reactant and product molecules in the gap

Considering a facet size of w = 37 nm for a NP with diameter of D = 80 nm ($f = w/D$, f = 0.456),[3] we obtain a total of ≈4,900 molecules adsorbed within the gap on the fcc(111) surface using the usual c(4x2) surface adsorption superlattice structure of thiolates. Based on the SERS intensity ratio between reactant and product in Suppl. Table 2, we estimate the number of molecules arriving at the gap to react. From the DFT calculations, the Raman intensity of the NC-BPT 1572 cm^{-1} mode is 23% stronger than that of 4-BTP 1532 cm^{-1} mode. Assuming the total number of adsorbed molecules stays constant during the reaction, we obtain the number of 4-BPT and NC-BPT molecules within a gap as shown in Suppl. Table 1. Furthermore, since the number of molecules, N, is approximately proportional to the surface area, A, the radial diffusion length from the edge of the NP facet, d, to the unreacted molecules, can be estimated as follows.

$$\frac{N_{\text{NC-BPT}}}{N_{\text{4-BTP}}} = \frac{A_{\text{NC-BPT}}}{A_{\text{4-BTP}}} = \frac{w^2-(w-2d)^2}{(w-2d)^2},\ N = N_{\text{4-BTP}} + N_{\text{NC-BPT}} \qquad (3)$$

$$d = \frac{w}{2}\left(1-\sqrt{\frac{N_{\text{4-BPT}}}{N}}\right) \qquad (4)$$

Calculated radial diffusion lengths to the reaction region at the beginning and end of the reaction are presented in Suppl. Table 1, and schematically represented in Suppl. Fig. 17. Saturation times are calculated by estimating time for all 4-BPT molecules are consumed. (Suppl. Table 4)

Time (s)	Au on Au			Au on Pd			Pd on Au			Pd on Pd		
	4-BPT	NC-BPT	d (nm)	4-BPT	NC-BPT	d (nm)	4-BPT	NC-BPT	d (nm)	4-BPT	NC-BPT	d (nm)
0	4641	259	0.5	4443	457	0.9	3689	1211	2.4	3193	1707	3.5
300	4573	327	0.6	3765	1135	2.3	2921	1979	4.2	2772	2128	4.6
$\Delta N/\Delta t$ (molecules s^{-1})	0.2			2.3			2.6			1.4		
Saturation time (min)	341			33			24			38		

Suppl. Table 4 | Estimated number of adsorbed 4-BPT and NC-BPT molecules within each nanogap. Radial diffusion lengths (d) are listed.

Supplementary references

1. Arul, R. *et al.* Giant mid-IR resonant coupling to molecular vibrations in sub-nm gaps of plasmonic multilayer metafilms. *Light. Sci. Appl.* **11**, 281 (2022).

2. Grys, D. B. *et al.* Controlling Atomic-Scale Restructuring and Cleaning of Gold Nanogap Multilayers for Surface-Enhanced Raman Scattering Sensing. *ACS Sens.* **8**, 2879–2888 (2023).

3. Elliott, E. *et al.* Fingerprinting the hidden facets of plasmonic nanocavities. *ACS Photonics* **9**, 2643–2651 (2022).
